# Preparative Biocatalytic Synthesis of α-Ketomethylselenobutyrate—A Putative Agent for Cancer Therapy

**DOI:** 10.3390/molecules28176178

**Published:** 2023-08-22

**Authors:** Maksim V. Nikulin, Viktor V. Drobot, Yevgeniya I. Shurubor, Vytas K. Švedas, Boris F. Krasnikov

**Affiliations:** 1 Belozersky Institute of Physicochemical Biology, Lomonosov Moscow State University, Lenin Hills 1, Bldg. 40, Moscow 119991, Russia; nikulin000@mail.ru (M.V.N.); linux776@gmail.com (V.V.D.); 2 Centre for Strategic Planning of FMBA of the Russian Federation, Pogodinskaya St., Bldg. 10, Moscow 119121, Russia; eshurubor@cspmz.ru; 3 Faculty of Bioengineering and Bioinformatics, Lomonosov Moscow State University, Lenin Hills 1, Bldg. 73, Moscow 119991, Russia

**Keywords:** α-ketomethylselenobutyrate, organoselenium compounds, histone deacetylase inhibition, cancer therapy, L-amino acid oxidase, preparative biocatalytic synthesis

## Abstract

Biomedical studies of the role of organic selenium compounds indicate that the amino acid derivative of L-selenomethionine, α-ketomethylselenobutyrate (KMSB), can be considered a potential anticancer therapeutic agent. It was noted that, in addition to a direct effect on redox signaling molecules, α-ketoacid metabolites of organoselenium compounds are able to change the status of histone acetylation and suppress the activity of histone deacetylases in cancer cells. However, the wide use of KMSB in biomedical research is hindered not only by its commercial unavailability, but also by the fact that there is no detailed information in the literature on possible methods for the synthesis of this compound. This paper describes in detail the procedure for obtaining a high-purity KMSB preparation (purity ≥ 99.3%) with a yield of the target product of more than 67%. L-amino acid oxidase obtained from *C. adamanteus* was used as a catalyst for the conversion of L-selenomethionine to KMSB. If necessary, this method can be used as a basis both for scaling up the synthesis of KMSB and for developing cost-effective biocatalytic technologies for obtaining other highly purified drugs.

## 1. Introduction

Selenium, one of the most important trace elements for the normal functioning of the body, enters the human body only as part of food or nutritional supplements. This trace element is indispensable, as it takes part in many cellular processes being part of the antioxidant enzymes in mammals. In particular, it plays the role of a cofactor in enzymatic reactions, and is also part of some selenoproteins, such as the enzymes glutathione peroxidase or iodothyronine deiodinase [1]. Due to its antioxidant and anti-inflammatory properties, selenium is able to modulate cell survival and proliferation [2,3]. At the same time, it should be noted that both excessively high and low levels of selenium can be dangerous to human health. The recommended daily dose of selenium established by the World Health Organization (WHO) for adults is about 55 µg per day [4]. Selenium deficiency occurs when it is taken with food in doses of less than 40 µg/day, which can provoke the development of heart, muscle disorders and joint disease [5,6]. At the same time, the upper safe level of selenium intake is considered to be a dose of up to 400 µg/day, although the maximum allowable doses of selenium intake are very individual and can vary from 600–900 to 1500–1600 µg/day [5,6]. An overdose of selenium (~5 mg per day) can cause the disruption of the normal biochemical functioning of the body, accompanied by hair loss, skin and nail damage, myocardial infarction, or kidney failure, as well as the development of a number of neurological disorders [7,8,9]. Post-mortem blood selenium levels due to acute selenium poisoning were above 1400 µg/L [10]. Rising mortality from cancer is driving the search for increasingly effective therapeutic agents and treatments for patients. Se compounds, which can be of organic, inorganic, natural, or synthetic origin, attract attention as promising drugs for the treatment of cancer. As a rule, such compounds target various molecular targets that contribute to the suppression of tumor cell growth [11]. Selenite and selenate can be distinguished among inorganic compounds, and selenides, diselenides, selenoethers, methylselenic acid, selenoamino acid, selol, and others among organic compounds. Most of these compounds are metabolized to form a number of redox compounds that differ in both the mechanism of action and toxicity, and the limit of doses used [12,13,14]. The toxicity of Se compounds largely depends on their chemical formula and dose, while inorganic analogues of Se compounds are usually more toxic than organic ones, and the concentration range of Se entering cells between insufficient and excess is quite narrow [12,15,16]. The anticancer effect of Se compounds is often due, for example, to the induction of apoptosis in cells, changes in gene expression, cell signaling pathways, effects on DNA, the process of tumor metastasis, and others [17,18]. Unfortunately, the mechanisms of action of Se compounds on the cell are not fully understood, especially since, depending on the dose, they can show opposite, both antioxidant and prooxidant properties [13].

A feature of Se is its high chemical/physical similarity to S, and the metabolic pathways of Se and S compounds are therefore often comparable. There is an assumption that Se can replace S in amino acids and other metabolites; however, a complete replacement of S still does not occur due to the higher reactivity of Se, which can also indirectly affect the manifestation of its toxicity [19,20]. As shown earlier, the toxicity of Se increases when it is absorbed by the cell under conditions of sulfate deficiency [20]. Compared to sulfur, selenium is more easily oxidized and kinetically more labile. In addition, while S chemistry is regulated in vivo, Se chemistry is not [21]. The toxicity of Se compounds towards cancer cells is due to a decrease in their proliferative properties associated with the inhibition of sulfhydryl groups. In addition, Se compounds, in particular a metabolite such as methylseleninic acid (MSA), can cause an increase in the generation of ROS by cancer cells, and lead to a decrease in intracellular glutathione, the appearance of a more oxidized environment and subsequent inhibition of cancer cells [22]. The role of glutathione in the resistance of cancer cells to a number of antitumor drugs was noted, which occurs due to the covalent binding of glutathione to them and inactivation of drugs [23]. The importance of glutathione for the metabolism of Se compounds has been shown in human lung cancer cells (line A549); it has been suggested that glutathione may play a key role in cell cycle arrest and Se (particularly MSA) induced apoptosis [24,25].

When studying the chemoprophylactic features of selenium, the main focus is on its ability to have a modulating effect on the onset and development of oncological diseases. This property of selenium is mediated by its antioxidant effect on normal cells, which leads to an increase in their ability to withstand oxidative stress [26]. However, selenium-containing compounds have a high prooxidant efficiency and selectivity for cancer cells, which is of particular interest to them as potential antitumor therapeutic agents [27,28].

Inorganic and organic derivatives of selenium and derivatives of the amino acids Se-methylselenocysteine and selenomethionine are also considered to be probable antitumor agents [14,29]. However, if inorganic selenium compounds are toxic and can cause a genotoxic effect, systemic toxicity, or the risk of metastatic formations, then its organic derivatives retain high antitumor activity and the ability to prevent cancer metastasis, and have fewer side effects and less pronounced systemic effects [30,31]. Organoselenium compounds have a pronounced specificity for cancer cells, which is expressed in their selective uptake and accumulation by cancer cells [14]. There are various approaches to inhibiting the growth of cancer cells using selenium-containing compounds. One of such promising areas is selenium nanoparticles under development. Selenium nanoparticles have selective cytotoxicity and can lead to the death of cancer cells without affecting normal cells. The important role of selenium nanoparticles in improving the functioning of the immune system, neurological and oncological diseases of various nature has been proven [32].

Another important approach to the treatment of cancer is the search and development of targeted selenium-containing inhibitors. This relatively new class of anticancer drugs includes histone deacetylase (HDAC) inhibitors, which are able to induce apoptosis and induce cell cycle arrest in cancer cells [29]. This group of substances includes α-ketomethylselenobutyrate (KMSB), which structurally resembles short-chain fatty acids (for example, butyric acid) and is an HDAC inhibitor [33]. It has been noted that KMSB exhibits a dose-dependent inhibitory activity against HDAC in leukemia, in human colon, prostate, breast and lung cancer cells [34,35,36]. Thus, experiments performed on purified human colon cancer cells revealed relative inhibition of HDAC1 and HDAC8 activity by KMSB supplementation. KMSB added to cancer cells in a concentration ranging from 0.02 to 0.2 and 2.0 mM showed that at the highest concentrations of KMSB, HDAC1 activity was reduced relative to the control by almost 20% (from 1.0 to <0.8) and HDAC8 activity was reduced by almost 40% (from 1.0 to <0.6) [34]. Studies conducted on lysates of androgen-sensitive LNCaP cells and androgen-independent LNCaP, C4-2, PC-3 and DU145 prostate cancer cells showed that KMSB at a concentration of 0.025 mM did not have a pronounced inhibitory effect, while at concentrations of 0.25 and 2.5 mM, significantly inhibited HDAC activity, which varied from 1.0 to ≥0.3–0.4 [35]. Interestingly, selenomethionine at concentrations up to 2–2.5 mmol/L did not have a significant effect on the inhibition of HDAC activity [34,35].

However, the commercial unavailability of KMSB does not allow for its wide application in the field of cancer therapy research, and there are no known methods for the preparative synthesis of KMSB in the scientific or patent-related literature.

In this work, we present a procedure for an optimized one-step synthesis of KMSB with a purity of ≥99.3% and a yield of the target product of ≥67% obtained from L-selenomethionine catalyzed by L-amino acid oxidase from the venom of the rattlesnake *Crotalus Adamanteus*. The method of KMSB synthesis developed will enable interested researchers to obtain a highly pure KMSB preparation in the laboratory for a detailed study of its antitumor properties and conduct clinical trials on models of various types of oncological diseases.

## 2. Results

**Figure 1:**
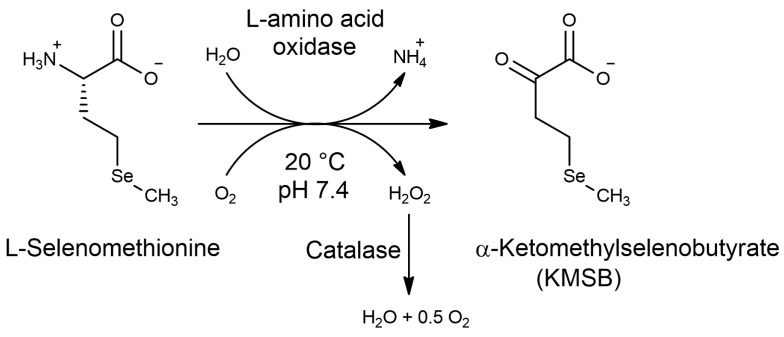
Scheme of biocatalytic synthesis of α-ketomethylselenobutyrate.

As a prototype for the development of the preparative synthesis of KMSB, we chose the previously described method for the preparation of α-ketoglutaramate (α-KGM), which consists of the oxidation of L-glutamine to α-KGM by L-amino acid oxidase in the presence of catalase [37]. We suggested that L-amino acid oxidase from *C. adamanteus* can also catalyze the oxidation of L-selenomethionine with atmospheric oxygen (Figure 1). In this case, according to the previous protocol, in order to avoid the occurrence of side processes, the resulting hydrogen peroxide can also be removed using catalase.

Since L-selenomethionine has a rather high cost (~1000 Euro/g), we performed a series of preliminary experiments with the more affordable amino acid L-methionine. We have tested variants of different temperatures of the reaction solution, initial concentrations of the substrate and enzymes, and methods of aeration of the solution with air. Experiments have shown that under various reaction conditions, the biocatalytic oxidation of L-methionine in the presence of L-amino acid oxidase from *C. adamanteus* and catalase leads to the formation of a significant amount of by-products. This was probably due to the strong reducing properties of L-methionine and α-ketomethylthiobutyrate, as well as catalase inhibition due to the possibility of binding L-methionine and α-ketomethylthiobutyrate through the sulfur atom in the coordination environment of the iron atom in the heme of catalase. As a result, the activity of catalase decreased during the reaction and this could lead to the accumulation of hydrogen peroxide in the reaction solution. In further experiments, our experience showed that the choice of L-methionine was not the most successful decision, since the behavior of L-selenomethionine in such biocatalytic systems differs significantly from that of L-methionine, and for the better. Thus, it was found that from a chemical point of view, L-methionine is not a complete analogue of L-selenomethionine (due to a significant difference in their chemical properties).

**Figure 2:**
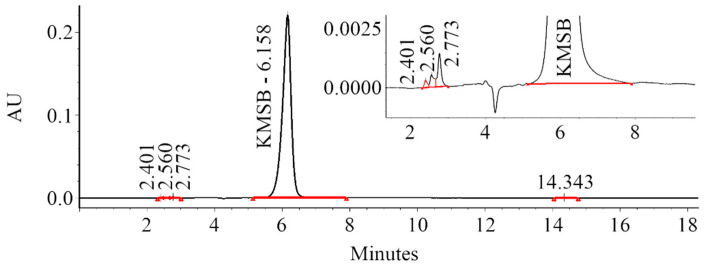
Chromatogram of the synthesized α-ketomethylselenobutyrate (HPLC-system: Waters 1525 Binary HPLC Pump, Waters 2489 UV/Visible Detector, Waters 2707 Autosampler; analysis conditions are specified in Section 4.3. Retention times: L-selenomethionine—3.28 min (not detected), α-ketomethylselenobutyrate—6.16 min (99.3%), unidentified impurities—2.40, 2.56, 2.77, 14.34 min (total 0.7%).

A biocatalytic synthesis of KMSB (Figure 1) was carried out from a 0.1 M aqueous solution of L-selenomethionine. The kinetics of formation of the target compound and by-products was monitored by HPLC. To this end, optimal conditions for chromatographic analysis were selected to ensure reliable determination of the components of the reaction mixture during the reaction time (see Section 4.3—Monitoring the progress of a biocatalytic reaction). After completion of the reaction and purification of the target product, the KMSB preparation was obtained in the form of a highly concentrated aqueous solution of sodium salt. Including the isolation and purification steps, the total yield of KMSB was over 67% and the chemical purity, according to HPLC data, was over 99.3%. Figure 2 shows the complete absence of the original L-selenomethionine in the resulting KMSB preparation.

**Figure 3:**
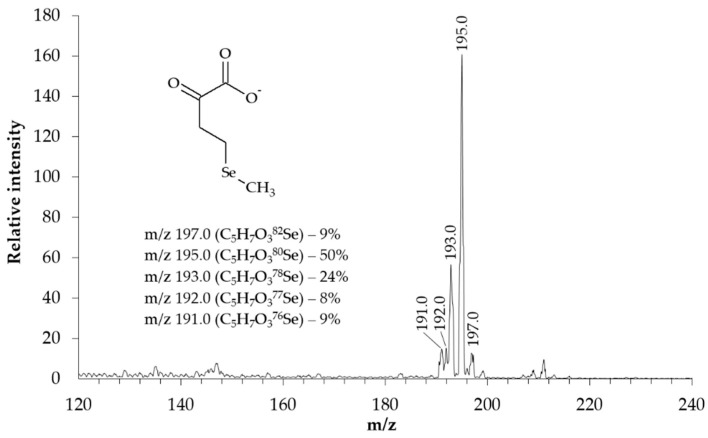
MS spectrum of the synthesized preparation of α-ketomethylselenobutyrate (3200 Q TRAP LC/MS/MS System, Applied Biosystems, triple quadrupole). Concentration of KMSB was 10 μM. Solvent–acetonitrile (LC/MS)/water MQ (1:1), direct infusion. Electrospray ionization (ESI-MS), ionization temperature 400 °C. Detection mode–negative ions. IonSpray Voltage 4150 V, declustering potential 15 V, entrance potential 6 V. The α-ketomethylselenobutyrate peaks correspond to the characteristic isotopic distribution of selenium.

**Figure 4:**
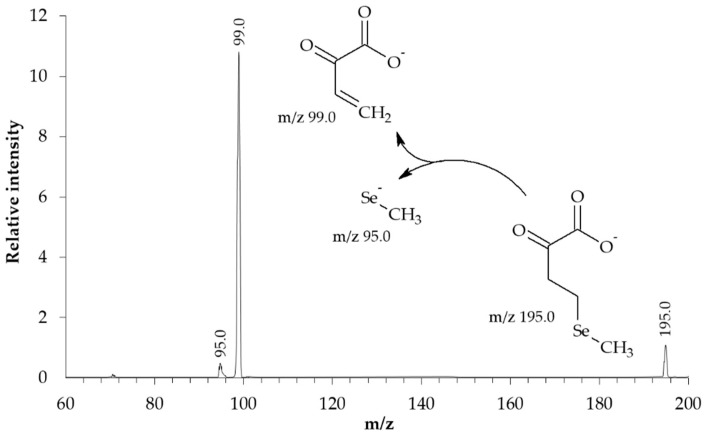
MS-MS spectrum of the synthesized preparation of α-ketomethylselenobutyrate (3200 Q TRAP LC/MS/MS System, Applied Biosystems, triple quadrupole). Concentration of KMSB was 10 μM. Solvent–acetonitrile (LC/MS)/water MQ (1:1), direct infusion. Electrospray ionization (ESI-MS), ionization temperature 400 °C. Detection mode–negative ions. Precursor ion–m/z 195.0. IonSpray Voltage 4150 V, declustering potential 15 V, entrance potential 6 V, collision energy 10 eV. Ion peak identification: α-ketomethylselenobutyrate (m/z 195.0), 2-oxobut-3-enoate (m/z 99.0), methylselenolate (m/z 95.0).

The structure of the synthesized KMSB was confirmed by MS. In the molecular ion detection mode, the MS spectrum (Figure 3) contains a series of signals in the region m/z 191–197. In this case, the signal intensity distribution corresponds to the characteristic isotopic distribution of selenium-containing compounds. Fragments of the dominant peak m/z 195.0 were analyzed in tandem MS/MS mode. On the MS/MS spectrum (Figure 4), in addition to the signal of the precursor ion m/z 195.0, the following signals of the corresponding fragments were identified: 2-oxobut-3-enoate (m/z 99.0) and methylselenolate (m/z 95.0).

**Figure 5:**
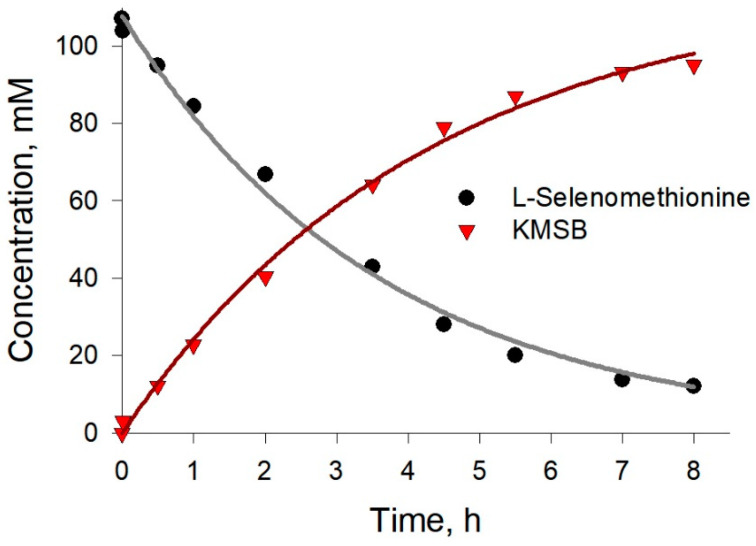
Representative kinetics of preparative production of α-ketomethylselenobutyrate by *C. adamanteus* L-amino acid oxidase-catalyzed reaction (20 °C, pH 7.4, 5 mg/mL) in the presence of catalase (0.4 mg/mL).

The developed HPLC method for determining the components of the reaction mixture made it possible to trace the kinetics of L-selenomethionine consumption and KMSB formation during the biocatalytic conversion (Figure 5).

**Figure 6:**
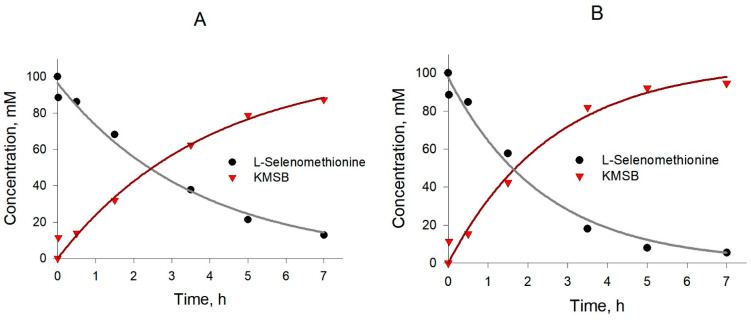
Kinetics of production of α-ketomethylselenobutyrate by *C. Adamanteus* L-amino acid oxidase-catalyzed reaction (pH 7.4, 5 mg/mL) in the presence of catalase (0.4 mg/mL) at different temperatures. (**A**) 10 °C; (**B**) 20 °C.

Experience with KMSB has shown that this compound is not very stable; therefore, it was important to optimize the temperature at which the enzymatic synthesis is carried out. The reaction was carried out according to the procedure described in Section 4.2. The preparation of KMSB took place at temperatures of 10 °C, 20 °C and 37 °C, while the volume of the reaction mixture was 1 mL. Kinetic curves under the respective conditions are presented in Figure 6. Other things being equal, after 7 h of reaction at 10 °C, the yield of KMSB reached 87%, and at 20 °C, it reached 95%. At the same time, the amount of accumulated impurities at temperatures of 10 °C and 20 °C turned out to be equal and contained to ~7% with respect to the accumulated amount of KMSB. At the same time, with an increase in the synthesis temperature to 37 °C, by-products in the reaction accumulated much faster, while the yield of KMSB in 7 h was no more than 50%. Thus, 20 °C was taken as the optimum temperature for the preparative synthesis of KMSB.

According to the scheme of biocatalytic synthesis of α-ketomethylselenobutyrate (Figure 1), a by-product of the enzymatic conversion of L-selenomethionine to KMSB is hydrogen peroxide, which must be immediately removed from the reaction mixture to avoid the accumulation of oxidation products of the selenium-containing reaction components, as well as to avoid enzyme inactivation. For this purpose, catalase is added to the reaction mixture at the optimal concentration we have established, about 0.4 mg/mL.

As part of the search for optimal conditions for the biocatalytic reaction, the initial concentration of L-selenomethionine was varied. When carrying out the enzymatic reaction with an initial concentration of L selenomethionine of 200 mM and a temperature of 25 °C, a significant slowdown in the reaction rate was observed, most likely associated with the inactivation of L-amino acid oxidase. At the same time, the output of KMSB for 23 h did not exceed 32%, and the amount of accumulated impurities exceeded 20%. Thus, it was decided to reduce the initial concentration of L-selenomethionine to approximately 100 ± 10 mM. In this case, other things being equal, the kinetic yield of KMSB increased to 85–90% in the 8 h of the reaction.

The mode of aeration of the reaction mixture, which was influenced by the shape of the vessel in which the reaction took place, was also of great importance. When the round-bottom flask was replaced by a flat-bottom flask with baffles, the KMSB yield increased by ~10–15%, and the impurity content decreased from ~10% to ~7%. In this case, the speed of the rocking chair was regulated in such a way as to prevent foaming of the reaction mixture. The best flask to reaction volume ratio for good aeration has been empirically determined to be 1:20–1:50. Thus, in the case of using a flat-bottom flask with baffles with a capacity of 500 mL, the optimal volume of the reaction mixture ranged from 10 to 25 mL at a rotation speed of 120 rpm. It should be noted that at the beginning of the reaction, with the addition of oxidase, a sharp accumulation of KMSB was observed in an amount of ~5–10% of the initial concentration of L-selenomethionine. Apparently, this was due to the rapid consumption of oxygen dissolved in water. Probably, the rate of further accumulation of KMSB was to some extent determined by the rate of diffusion of oxygen from the air into the reaction mixture; therefore, the choice of the aeration mode was essential.

Thus, the optimal conditions for the enzymatic reaction included the following parameters: initial concentration of L-selenomethionine for preparative synthesis was 100 ± 10 mM, the concentration of L-amino acid oxidase was about 5 mg/mL, the catalase concentration was 0.4 mg/mL, the temperature was 20 °C, the volume of the reaction mixture was from 10 to 25 mL, and the synthesis time was about 7–8 h. Under these conditions, the concentration of enzymes did not affect the ratio of accumulated KMSB and impurities. However, with a decrease in the concentration of enzymes, a regular decrease in the reaction rate occurred, while non-enzymatic processes could lead to a significant accumulation of impurities.

The procedure for the purification of the KMSB preparation after the enzymatic reaction consisted of the deproteinization of the reaction mixture, its purification on an ion exchange resin, followed by extraction with an organic solvent. Deproteinization was performed by ultrafiltration through centrifuge filters with a pore size that cuts off proteins with a molecular weight of more than 30 kDa. It should be noted that no significant decrease in enzyme activity was observed during the reaction; thus, the remaining solution with enzymes could be reused. KMSB was purified from unreacted L-selenomethionine, ammonium ions, and metal cations on a Dowex cation exchange resin column using isocratic elution with distilled water. In the collected KMSB fraction, no residual L-selenomethionine was detected by HPLC. Thus, the method presented permitted us to completely remove the residues of L-selenomethionine. Extraction of KMSB with an organic solvent, in particular, methylene chloride, from an aqueous solution with a pH below 2 made it possible to purify KMSB from unidentified impurities and obtain a product with a chemical purity of more than 99.3%. The total yield of the KMSB (taking into account the stages of isolation and purification) was 50–70%. Table S1 in Supplementary Materials shows the results of biocatalytic reactions for the synthesis of KMSB according to the above procedure. These data demonstrate how the developed methodology works in practice in the routine laboratory preparation of KMSB in preparative amounts. The high yields and high chemical purity of the obtained KMSB in a series of experiments indicate the reproducibility and robustness of the method.

The concentration of the final KMSB solution depended on the degree of dilution of the oily residue obtained after synthesis and purification, and could be up to 0.5 M. The experiments showed that the KMSB preparation in its pure form, without solvents, is not stable at room temperature, rapidly oxidizes and decomposes. Therefore, it is recommended to store concentrated KMSB solutions at pH ~6, frozen, at a temperature not exceeding −20 °C.

## 3. Discussion

Selenium deficiency correlates with an increase in cancer and mortality, while the intake of selenium supplements reduces this risk [38,39]. In this regard, interest in natural and synthetic organoselenium compounds as potential anticancer agents is growing. It has been shown that organoselenium compounds can act as inhibitors of histone deacetylase, and alter the acetylation of histones and nonhistone proteins, thereby regulating gene and protein expression [34,35]. 

The regulation of oxidative stress in cells is an important factor in both tumor development and anticancer therapy. There are a number of signaling pathways and mechanisms that affect the metabolism of reactive oxygen species (ROS) [40]. The redirection of ROS metabolism to achieve a critical state for cancer cells while maintaining functions in normal cells can be considered the defining task here [41].

It has been established that selenium compounds, which have high selectivity and sensitivity, are able to modulate the redox potential in cancer cells [42]. The chemoprophylactic and therapeutic activity of organoselenium compounds is due to certain changes in the epigenome and is associated with histone acetylation [34,35]. HDACs are actively involved in the regulation of gene expression; therefore, determining the range of their potential inhibitors becomes an important therapeutic task in the treatment of tumor diseases. It was previously mentioned that organoselenium compounds can act as HDAC inhibitors by altering histone acetylation, regulating gene expression and protein activity [34,35]. It was shown that Se-methylselenocysteine and L-selenomethionine, which are substrates of glutamine transaminase K (GTK) and L-amino acid oxidase, are also able to inhibit HDAC [43]. It has been noted that GTK and L-amino acid oxidase convert Se-methylselenocysteine to β-methylselenopyruvate, while L-amino acid oxidase converts L-selenomethionine to KMSB. Structurally, these metabolites resemble butyrate, which is known as an HDAC inhibitor. It was found that β-methylselenopyruvate and KMSB inhibit HDAC activity to a much greater extent than Se-methylselenocysteine or L-selenomethionine [35].

The study of the anticancer properties of HDAC inhibitors, and their possible use for therapeutic needs, requires the development of effective methods for the synthesis of selenium-containing α-keto acids.

As a result of studying the metabolism of L-selenomethionine derivatives using mass spectrometry (MS) and HPLC, it was shown that KMSB is formed in situ upon the interaction of L-amino acid oxidase with L-selenomethionine [34,35,44]. This observation led to the development of a methodology for determining the content and conversion of KMSB in living systems. The possibility of conversion of L-selenomethionine and formation of KMSB was also shown using kynurenine aminotransferase III and glutamine transaminase L [43]. A method for the preparation of a structural analog of KMSB, 2-hydroxymethyl selenobutyrate, was described, and the idea of the possibility of obtaining KMSB using a biocatalytic oxidation of 2-hydroxymethyl selenobutyrate in the presence of an enzyme of the oxidoreductase class was put forward [45]. However, any method for the synthesis of KMSB or the determination of a specific enzyme capable of catalyzing such a reaction is not presented in the scientific literature. Perhaps, this is due to the complexity of experimental work with pungent and reactive selenium compounds, as well as the difficulty of their isolation and subsequent purification.

Thus, as a result of this study, an effective method for the biocatalytic synthesis of KMSB in the form of a highly concentrated solution of sodium salt with a minimum content of impurities was developed. The use of enzymes as a catalyst, as well as optimization of reaction conditions and purification of the final product, made it possible to synthesize KMSB in high yield (≥67%) and high chemical purity ≥ 99.3%.

## 4. Materials and Methods

### 4.1. Reagents

All reagents used were of the highest quality. L-selenomethionine (≥98% (TLC), powder), amino acid L-oxidase from lyophilized *C. Adamanteus* venom (type I, dried venom, 0.3 U/mg, solid), catalase (from lyophilized bovine liver powder, 2000–5000 U/mg protein), and cation exchange resin (H^+^-form of Amber-Chrom (previously Dowex) 50WX4) were obtained from Sigma-Aldrich Chemical, Saint Louis, MO, USA.

### 4.2. Synthesis of KMSB

A portion of L-selenomethionine (500 mg) was dissolved in 22 mL of distilled water, and the pH of the solution was adjusted to 7.4 with 1 M NaOH. The solution was transferred to a 0.5-L flat-bottomed conical flask with 4 baffles; 10 mg of catalase was added to remove the formed hydrogen peroxide and a solution (~3 mL) containing 125 mg of L-amino acid oxidase from *C. Adamanteus*, which was obtained by pre-dialysis against distilled water. For better aeration, the flask was loosely covered with perforated aluminum foil. The mixture was incubated in a thermostatted shaker at 20 °C with constant stirring at 120 rpm. The shaker with the flask was placed in a fume hood operating at maximum power, and the reaction was carried out for 8 h.

### 4.3. Monitoring the Progress of a Biocatalytic Reaction

To monitor the progress of the reaction, a 30 μL aliquot was taken from the reaction mixture and mixed with 970 μL of 67% aqueous acetonitrile solution and the protein was precipitated. The solution was centrifuged for 3 min at 10,000× *g*. A 150 µL aliquot of the centrifuged mixture was diluted with 850 µL of buffer solution (20 mM KH_2_PO_4_, pH 2.9) and analyzed by high performance liquid chromatography (HPLC). HPLC analysis conditions were as follows: AkzoNobel Kromasil Eternity-5-C18 column, 4.6 × 250 mm, mobile phase 1:9 (*v*/*v*%) acetonitrile/20 mM KH_2_PO_4_ buffer pH 2.9; flow rate was 1 mL/min; the injected sample volume was 20 μL; the column temperature was 25 °C; and UV detection occurred at 210 nm. The retention time on the column for L-selenomethionine was 3.28 min, and for KMSB, it was 6.16 min.

### 4.4. Isolation, Purification and Concentration of KMSB

Upon completion of the procedure for obtaining KMSB, the reaction mixture was centrifuged for 30 min at 12,000× *g*. The supernatant was deproteinized by ultrafiltration using centrifugal filters (Amicon Ultra-15, cutoff 30 kDa, Merck Millipore, Ireland). Ultrafiltration was carried out at 4 °C and 4000 g for 20–25 min. The enzyme concentrate obtained as a result of this procedure can be reused. The collected deproteinized solution was purified from unreacted L-selenomethionine by ion exchange chromatography on a column with Dowex cation exchange resin in H^+^-form. The column parameters were as follows: length 40 cm, diameter 20 mm, and holding temperature 4 °C. The eluent was passed through the column using a high pressure pump at a rate of 6 mL/min. The ion exchange resin was preliminarily activated by sequential washing with 500 mL of bidistilled water, 100 mL of 1 M HCl, and 500 mL of bidistilled water until the aqueous eluate became neutral (pH 6.0–6.5). The deproteinized solution was then loaded onto a Dowex activated resin column in H^+^-form. The mixture was eluted with bi-distilled water at a rate of 6 mL/min. The eluate fractions were collected in a volume of 4 mL. Fractions containing KMSB were identified by their pH using indicator paper (pH < 4).

The fractions were then combined, and the pH was adjusted to 1.9 with 37% hydrochloric acid. KMSB was extracted from the aqueous phase with an equal volume of methylene chloride at least 10 times. Then, the solvent was removed on a rotary evaporator, and the oily residue was dissolved in water to obtain the desired concentration. At the same time, the pH of the solution was adjusted to 6.4 with 5 M NaOH. Upon completion of the synthesis procedure, the purity of the obtained sodium KMSB, according to HPLC analysis, was about 99.3%. The overall yield of the reaction, taking into account purification and concentration, was ≥67%. 

The structure of the synthesized KMSB was confirmed by MS spectroscopy in molecular ion detection mode on a 3200 Q TRAP LC/MS/MS System, Applied Biosystems, triple quadrupole. The concentration of KMSB was 10 μM. Solvent–acetonitrile (LC/MS)/water MQ (1:1), direct infusion, flow 0.1 mL/min. Electrospray ionization (ESI-MS), ionization temperature 400 °C. Detection mode–negative ions. IonSpray Voltage 4150 V, declustering potential 15 V, entrance potential 6 V. KMSB degradation products were identified in MS/MS mode on the same system. The concentration of KMSB was 10 μM. Solvent–acetonitrile (LC/MS)/water MQ (1:1), direct infusion, flow 0.1 mL/min. Electrospray ionization (ESI-MS), ionization temperature 400 °C. Detection mode–negative ions. Precursor ion–m/z 195.0. IonSpray Voltage 4150 V, declustering potential 15 V, entrance potential 6 V, collision energy 10 eV. Ion peak identification: α-ketomethylselenobutyrate (m/z 195.0), 2-oxobut-3-enoate (m/z 99.0), methylselenolate (m/z 95.0).

## 5. Conclusions

The development of an effective method for obtaining KMSB was due to the need to conduct planned scientific and clinical trials to identify the prospects for the use of KMSB as an antitumor therapeutic agent. If its therapeutic status is confirmed, the technologies developed can be used for a larger and more cost-effective synthesis of the KMSB preparation.

As a result of the experimental work, the amount of the KMSB preparation required for future studies was synthesized, and the conditions for its isolation from the solution, purification and storage methods were worked out. The kinetics of accumulation of KMSB from L-selenomethionine catalyzed by L-amino acid oxidase from *C. Adamanteus* in the presence of catalase was studied. The importance of choosing the optimal conditions for carrying out the reaction, ensuring an optimal aeration of the solution for a sufficient supply of atmospheric oxygen to the reaction mixture, is emphasized. As a result, the KMSB preparation was obtained as a solution with an overall yield of ≥67% and a chemical purity of ≥99.3%. The structure of the synthesized KMSB preparation was confirmed by MS.

## 6. Patents

The authors decided to patent the described method for the synthesis of KMSB. Application № 2021121230 for a patent for an invention was filed on 19 July 2021 with the Federal Service for Intellectual Property of the Russian Federation (FIPS RF). The patent for invention № 2776282 was issued by the FIPS RF on 15 July 2022. The name of the invention is “Method for the synthesis of alpha-ketomethylselenobutyrate”.

## Data Availability

Additional data can be available upon personal request.

## Supplementary Materials

The following supporting information can be downloaded at: https://www.mdpi.com/article/10.3390/molecules28176178/s1, Table S1. The results of the performed biocatalytic reactions for the synthesis of KMSB.

## Sample Availability

Samples of KMSB can be available from the authors by personal request.
