# Supplementary material for: Preparative Biocatalytic Synthesis of α-Ketomethylselenobutyrate—A Putative Agent for Cancer Therapy
Source: Molecules. 2023 Aug 22;28(17):6178. doi: 10.3390/molecules28176178 (PMC10489025; doi:10.3390/molecules28176178)
Supplement: Supplementary file 1 [file molecules-28-06178-s001.zip › molecules-2509078-SI.pdf]

Supplementary materials

Table S1. The results of the performed biocatalytic reactions for the synthesis of KMSB.

**Experimental Stage**	**Experiment number**			
	**1**	**2**	**3**	**4**
Initial concentration of L-selenomethionine, mM	102	110	107	103
Reaction mixture volume, ml	13	10	25	25
Concentration of L-amino acid oxidase, mg/ml	5	5	4.6	4.2
Concentration of catalase, mg/ml	0.4	0.4	0.4	0.4
Reaction time, h	7.5	6.9	8	7.5
Yield of the KMSB by kinetics, %	88	80	90	87
Total yield of the KMSB (taking into account the stages of isolation and purification), %	60	51	64	67
Chemical purity of the KMSB, %	99.3	99.5	99.6	99.6
Content of L-selenomethionine in the KMSB preparation, %	not detected	not detected	not detected	not detected
Content of unidentified impurities in the KMSB preparation, %	0.7	0.5	0.4	0.4
The volume of the final solution of the KMSB, ml	3.5	2.5	7	6.5
Concentration of KMSB in the final solution, mM	230	220	240	270
